# Flexible Random Laser Using Silver Nanoflowers

**DOI:** 10.3390/polym11040619

**Published:** 2019-04-03

**Authors:** Junhua Tong, Songtao Li, Chao Chen, Yulan Fu, Fengzhao Cao, Lianze Niu, Tianrui Zhai, Xinping Zhang

**Affiliations:** 1 Institute of Information Photonics Technology and College of Applied Sciences, Beijing University of Technology, Beijing 100124, China; jhtong@emails.bjut.edu.cn (J.T.); songtaoli2001@126.com (S.L.); s201706083@emails.bjut.edu.cn (C.C.); fuyl@bjut.edu.cn (Y.F.); wincfz@163.com (F.C.); niulianze@126.com (L.N.); zhangxinping@bjut.edu.cn (X.Z.); 2 A School of Mathematics & Physics, North China Electric Power University, Baoding 071003, China

**Keywords:** polymer waveguides, plasmonics, random laser, tunable

## Abstract

A random laser was achieved in a polymer membrane with silver nanoflowers on a flexible substrate. The strong confinement of the polymer waveguide and the localized field enhancement of silver nanoflowers were essential for the low-threshold random lasing action. The lasing wavelength can be tuned by bending the flexible substrate. The solution phase synthesis of the silver nanoflowers enables easy realization of this type of random lasers. The flexible and high-efficiency random lasers provide favorable factors for the development of imaging and sensing devices.

## 1. Introduction

Random lasers, characterized by low spatial coherence, easy preparation, and low cost [1,2,3], have attracted wide attention due to their potential applications in imaging [4], sensing [5] and integrated devices [6]. Until recently, various random lasers in both liquid [7,8] and solid state [9,10] have been studied. In comparison with liquid random lasers, solid-state random lasers are superior in optical confinement, packaging, stability, and repeatability, which promote additional applications. Flexible random lasers also have achieved considerable progress in the tunability and flexibility of solid-state random lasers [11,12]. Various nanostructures have been introduced as scattering centers in flexible random lasers, such as ZnO nanoparticles [13,14], graphene structures [15], carbon dots [16], and metal nanostructures [17,18]. However, the output performance of flexible random lasers, such as the threshold, needs to be further optimized. 

Metal nanostructures are favorable in the obtainment of low-threshold random lasing action because they can provide localized surface plasmon resonance and increase the effective cross-section for multiple light scattering [19,20,21]. Among metal nanostructures, silver nanoflowers (Ag NFs), which provide an abundance of nanogaps and spiky tips, have been widely used in surface-enhanced Raman scattering [22,23,24]. Recently, high-performance random lasing covering visible region was achieved by doping Ag NFs in a dye solution [25]. However, solid-state random lasers using Ag NFs have been rarely reported. Accordingly, to improve the performance of flexible random lasers, it is necessary to explore effective methods to design a stable system using Ag NFs. 

In this paper, a flexible random laser was fabricated by attaching a polymer membrane on Ag NFs. Ag NFs were optimized in size and morphology using an ice-water bath. A random lasing action was observed at 566.7 nm with a low threshold of 6.1 μJ/cm^2^, which resorted to the localized field enhancement of Ag NFs. The wavelength of a random laser using Ag NFs can be tuned and restored by bending the flexible substrate. 

## 2. Fabrication Methods and Spectra Characterizations

Ag NFs were synthesized using the ice-water bath method [25]. AgNO_3_ (Aldrich, Fengxian, Shanghai, China), citric acid (Aladdin, Fengxian, Shanghai, China), PVP (polyvinylpyrrolidone, Aladdin, Fengxian, Shanghai, China), and ascorbic acid (Aldrich, Fengxian, Shanghai, China) were chosen as the oxidant, dispersant, blocking agent, and reductant, respectively. Four different solutions of AgNO_3_ aqueous (1 mL, 24 mM), PVP solution (1 mL, 15 mM), citric acid solution (0.1 mL, 12 mM), and ascorbic acid aqueous solution (C_6_H_8_O_6_, 1 mL, 24 mM) were successively added into the deionized water (10 mL) in an ice-water bath with magnetic stirring for 5 min, 10 min, 10 min, and 5 min, respectively, accompanied by a color change in the mixture from colorless to dark grey. The final reaction solution was centrifuged, re-dispersed, and stored in ethyl alcohol.

The free-standing polymer membrane was both flexible and transplantable and acted as an active layer for random lasers, which was prepared by the method reported previously [18,26,27]. Polyvinyl alcohol (PVA 107, Celanese Chemicals, Oberhausen, Nordrhein-Westfalen, Germany) aqueous solution (40 mg/mL) was spin-coated onto a polyethylene terephthalate (PET) substrate (20 × 15 × 1 mm) at a speed of 3000 rpm, forming a film with a thickness of 350 nm. Subsequently, the solution of a luminescent polymer, poly [(9,9-dioctylfluorenyl-2,7-diyl)-alt-co-(1,4-benzo-(2,1,3)-thiadiazole)] (F8BT, American Dye Source, Monteral, Quebec, Canada) with a concentration of 23.5 mg/mL in xylene, was spin-coated on the PVA film at a speed of 2500 rpm, forming a 200-nm-thick film. The prepared structure was then immersed in the deionized water to dissolve the PVA layer. The polymer membrane of F8BT was separated from the PET substrate and floated on the surface of water.

**Figure 1:**
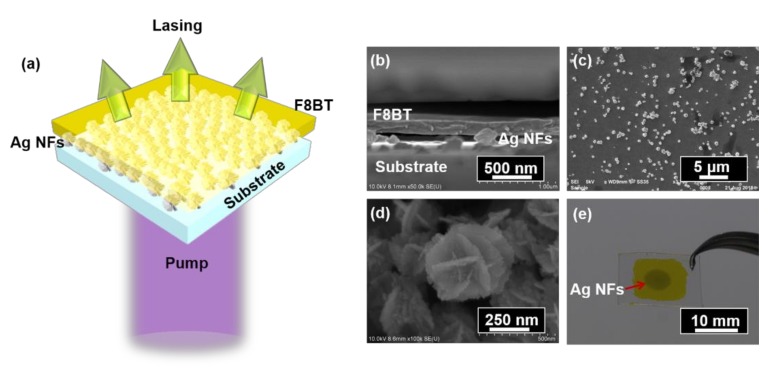
(**a**) Schematic of the proposed random laser. (**b**) Side-view SEM image of the random laser. (**c**) Top-view SEM image of the Ag NFs structure. (**d**) SEM image of Ag NFs at high magnification. (**e**) Photograph of the random laser. The red arrow indicates the Ag NFs structure.

The Ag NFs (4 mg/mL, 10 μL) suspension was dipped on the PET substrate and dried at room temperature for 2 min. The wet polymer membrane was then transferred to Ag NFs on the PET substrate. The F8BT membrane can stick tightly to Ag NFs after drying naturally at room temperature due to surface intension. Subsequently, a flexible plasmonnic random laser was achieved, as shown in Figure 1a.

The sample was vertically excited by a frequency-doubled pulse laser with a wavelength of 400 nm, a pulse duration of 200 fs, a repetition rate of 1 kHz, and an output beam diameter of 3 mm, as illustrated in Figure 1a. The intensity of the laser beam was tuned continuously by a variable optical attenuator. The emission spectrum was collected by a spectrometer (Maya 2000 Pro, Ocean Optics, Dunedin, FL, USA).

Figure 1b shows the scanning electron microscope (SEM) image of the sample from the side-view, illustrating that the polymer membrane with the thickness of 200 nm was well supported by the Ag NFs. The air gaps between the Ag NFs acted as a low-index spacer, strengthening the scattering effect. The top-view SEM image of the Ag NFs on PET substrate is shown in Figure 1c, indicating that the Ag NFs were distributed randomly on the substrate. In our experiment, the typical size of the Ag NFs ranged from 100 nm to 300 nm. The nanostructure of Ag NFs is characterized in Figure 1d, which shows that there were abundant nanogaps in each Ag NF and between adjacent Ag NFs. The polymer membrane was smoothly attached on the Ag NFs due to the surface tension effect, as shown in Figure 1e. The Ag NFs structure is denoted by a red arrow.

**Figure 2:**
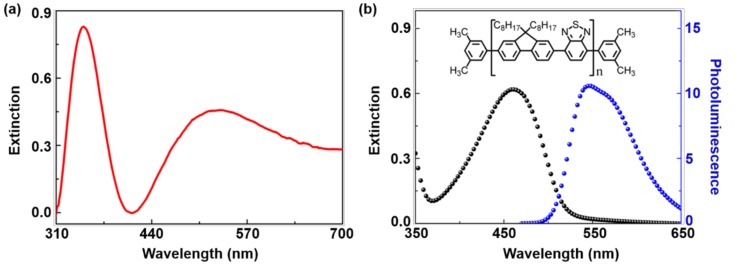
(**a**) Extinction spectrum of Ag NFs. (**b**) Extinction spectrum (black curve) and photoluminescence spectrum (blue curve) of F8BT membrane. Inset: the molecular structure of F8BT.

The extinction spectrum of Ag NFs in Figure 2a revealed a broadband ranging from 300 nm to 700 nm, with two distinct extinction peaks at λ = 350 nm, and λ = 532 nm, respectively. The peak at 350 nm corresponds to the high-order plasmon resonance, which was attributed to the sharp edges and spikes in the Ag NFs. The peak at 532 nm corresponded to the low-order (dipole) plasmon resonance [28]. Figure 2b shows the photoluminescence spectrum (blue curve) and extinction spectrum (black curve) of the F8BT membrane. The photoluminescence spectrum of the F8BT membrane overlaps well with the extinction spectrum of the Ag NFs in Figure 2a, indicating that the emission of F8BT could be enhanced by Ag NFs.

**Figure 3:**
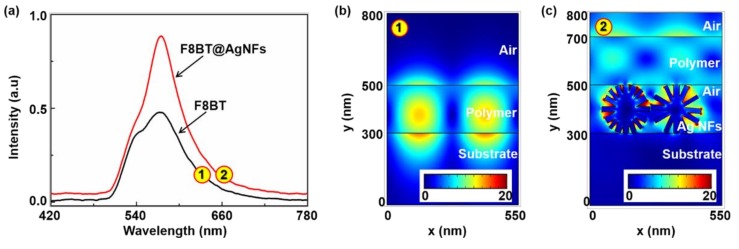
(**a**) Normalized photoluminescence spectra of F8BT membrane (black curve) and F8BT/Ag NFs film (red curve). Electric field distributions of (**b**) the polymer waveguide and (**c**) the F8BT/Ag NFs structure at 566.7 nm.

To understand the field enhancement effect of Ag NFs in such a polymer/Ag NFs/substrate configuration, experimental measurements and numerical simulations were performed, as shown in Figure 3. Figure 3a shows the normalized emission spectra of the F8BT film (black curve) and F8BT/Ag NFs (red curve). The enhanced emission intensity of F8BT/Ag NFs resulted from the localized surface plasmon resonance of Ag NFs, which enhanced the effective cross-section for multiple scattering processes.

The electric field distributions of F8BT film and F8BT/Ag NFs were simulated by the finite element method in COMSOL, as shown in Figure 3b,c. The non-periodic boundary above the device was added to avoid boundary conditions interfering with evanescent fields. The refractive indices of the F8BT and Ag were 1.94 and 0.05 + 3.858i, respectively. All refractive indices were measured by a spectroscopic ellipsometer (ESNano, Ellitop). For simplicity, the refractive index of Ag NFs was chosen to be the same as that of an Ag film (25 nm). The results demonstrate that strong localized field enhancement can be observed around the tips of Ag NFs, between “petals” of Ag NFs and adjacent Ag NFs, which are consistent with experimental results. The distribution of the waveguide modes are affected significantly by the Ag NFs. The Ag NFs not only provide the plasmonic enhancement but also extract the light from the waveguide by strong scattering.

**Figure 4:**
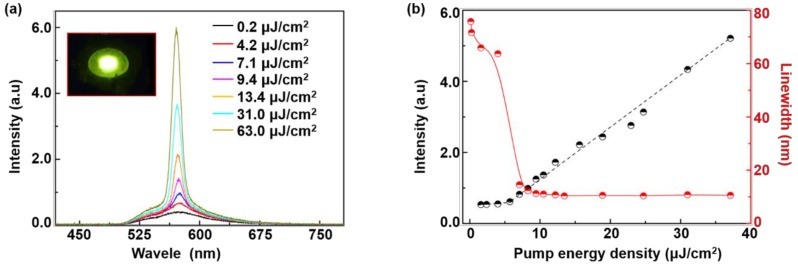
(**a**) Emission spectra of the random laser with different pump energy densities. Inset: photograph of the operating random laser. (**b**) Emission intensities (black dots) and the linewidth (red dots) as a function of the pump energy densities.

Figure 4a shows the emission spectra of the proposed random laser under different pump energy densities. Spontaneous emissions were observed at low pump energy densities (<6.1 μJ/cm^2^). By increasing the pump energy densities (≥6.1 μJ/cm^2^), there was a peak at 566.7 nm with the full width at half maximum (FWHM) of the emission peaks less than 10 nm, indicating the accomulation of random lasing. Bright yellow light could be observed in the photograph of the random laser, as shown in the inset of Figure 4a. Figure 4b shows the emission intensity and the FWHM of the random laser as a function of the pump energy densities, showing the threshold as low as 6.1 μJ/cm^2^. In comparison with our previous results [29,30], the low-threshold random lasing action is attributed to the strong plasmonic enhancement and high-quality waveguide confinement provided by the Ag NFs and the polymer membrane, respectively.

**Figure 5:**
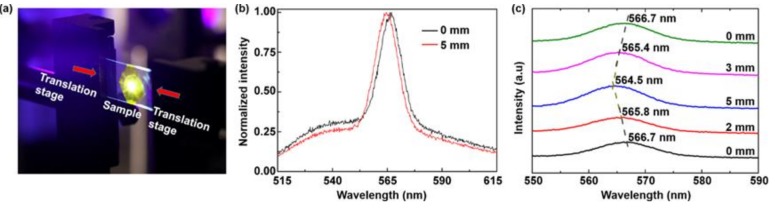
(**a**) Experimental set-up for compression (0 mm–2 mm–5 mm) and restoring (5 mm–3 mm–0 mm) processes. (**b**) Tuning of emission wavelength by bending the random laser. (**c**) Spectral evolution of the random laser under bending and recovering processes.

Furthermore, the wavelength tunability was demonstrated by bending the flexible sample, as shown in Figure 5. The PET substrate was bent into convex shapes by compressing the distance between the two translation stages. The direction of the compression was along the long axis (20 mm) of the substrate. The emission wavelength could be tuned by bending the random laser, as shown in Figure 5b. Figure 5c presents the evolution of the spectra for the flexible random laser with different amounts of compression. The corresponding emission wavelength was blue shifted in relation to the increase in compression. When the total compressing amount was 5 mm, a shift larger than 2 nm was observed. The blue-shift of emission wavelength was attributed to the increase of inter-particle distances between Ag NFs, which altered the mutual plasmon interaction and the scattering ability of different emitted light. Additionally, the emission wavelength could be gradually restored when the strain was released to zero, which exhibited good repeatability.

## 3. Conclusions

A flexible random laser was achieved using Ag NFs and a polymer membrane. The sample was fabricated by an ice-water bath and a free-standing membrane method. The low threshold and high efficient performance of the random laser was assisted by the plasmonic enhancement of Ag NFs and the confinement of the polymer waveguide. The emission wavelength can be tuned by bending the flexible sample and restored by releasing the strain. The fabrication method is simple and low cost. These superior performances of the flexible random laser using Ag NFs could promote the application of random lasers.
